# Ferulic Acid Enhances Oocyte Maturation and the Subsequent Development of Bovine Oocytes

**DOI:** 10.3390/ijms241914804

**Published:** 2023-09-30

**Authors:** Yu Wang, Jia-Jia Qi, Yi-Jing Yin, Hao Jiang, Jia-Bao Zhang, Shuang Liang, Bao Yuan

**Affiliations:** Department of Animals Sciences, College of Animal Sciences, Jilin University, Changchun 130062, China; ywang20@mails.jlu.edu.cn (Y.W.); qijj20@mails.jlu.edu.cn (J.-J.Q.); yinyj21@mails.jlu.edu.cn (Y.-J.Y.); jhhaojiang@jlu.edu.cn (H.J.); zjb515@163.com (J.-B.Z.)

**Keywords:** bovine, oocyte quality, in vitro maturation, ferulic acid, oxidative stress

## Abstract

Improving the quality of oocytes matured in vitro is integral to enhancing the efficacy of in vitro embryo production. Oxidative stress is one of the primary causes of quality decline in oocytes matured in vitro. In this study, ferulic acid (FA), a natural antioxidant found in plant cell walls, was investigated to evaluate its impact on bovine oocyte maturation and subsequent embryonic development. Bovine cumulus–oocyte complexes (COCs) were treated with different concentrations of FA (0, 2.5, 5, 10, 20 μM) during in vitro maturation (IVM). Compared to the control group, supplementation with 5 μM FA significantly enhanced the maturation rates of bovine oocytes and the expansion of the cumulus cells area, as well as the subsequent cleavage and blastocyst formation rates after in vitro fertilization (IVF) and somatic cell nuclear transfer (SCNT). Furthermore, FA supplementation was observed to effectively decrease the levels of ROS in bovine oocytes and improve their mitochondrial function. Our experiments demonstrate that FA can maintain the levels of antioxidants (*GSH*, *SOD*, *CAT*) in oocytes, thereby alleviating the oxidative stress induced by H_2_O_2_. RT-qPCR results revealed that, after FA treatment, the relative mRNA expression levels of genes related to oocyte maturation (*GDF-9* and *BMP-15*), cumulus cell expansion (*HAS2*, *PTX3*, *CX37*, and *CX43*), and embryo pluripotency (*OCT4*, *SOX2*, and *CDX2*) were significantly increased. In conclusion, these findings demonstrate that FA supplementation during bovine oocyte IVM can enhance oocyte quality and the developmental potential of subsequent embryos.

## 1. Introduction

In vitro embryo production (IVEP) enables the generation of a substantial number of viable embryos, thereby shortening generation intervals and expediting the breeding process of bovines. However, IVEP techniques entail various drawbacks, such as the occurrence of developmental arrest during in vitro embryo development and low survival rates among offspring [1,2]. Numerous studies have shown that a significant decline in oocyte quality after in vitro maturation (IVM) is the primary underlying cause of these issues [3,4]. Oxidative stress has been found to have toxic effects on oocyte maturation and is considered one of the contributing factors to poor oocyte quality [5,6]. It can lead to functional damage to various organelles in oocytes, which negatively impacts oocyte quality and even leads to oocyte apoptosis [7,8,9]. Notably, oxidative stress can induce apoptosis in cumulus cells, thereby impairing their ability to exchange substances with oocytes. This can affect the nutritional supply of oocytes, ultimately resulting in a decrease in oocyte quality [10,11].

Excessive ROS production in oocytes is the primary cause of cellular oxidative stress in these cells [12]. During in vivo oocyte maturation, mitochondria produce ROS, which are involved in intracellular signaling and gene expression [13,14]. Excess ROS are rapidly broken down by antioxidant enzymes in the follicular fluid, maintaining a delicate balance between ROS generation and elimination in cells [15,16,17]. However, oocytes created by IVM lack the protection provided by these antioxidant enzymes, resulting in the breakdown of this balance and the accumulation of increasing numbers of ROS molecules. These molecules can attack other organelles, leading to cellular dysfunction such as endoplasmic reticulum stress, spindle abnormalities, and cytoskeletal damage, ultimately resulting in cell cycle arrest and apoptosis [9,18]. In an effort to mitigate the detrimental effects of oxidative stress on oocyte quality, researchers have explored the use of antioxidant supplements such as melatonin [19], asiatic acid [20], and resveratrol [21] in IVM medium to alleviate oxidative stress in order to enhance the quality of oocytes and subsequent embryonic development. These studies indicate that supplementation with antioxidants can improve the quality of oocytes and subsequent embryonic development.

**Figure 1:**
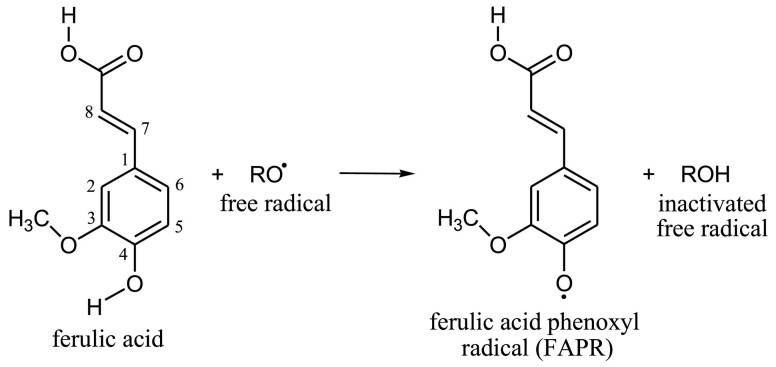
Formation of FAPR.

Ferulic acid (FA) is widely recognized as an excellent natural antioxidant that functions as a free radical scavenger, an inhibitor of catalytic free radical-generating enzymes, and an enhancer of scavenger enzyme activity [22,23]. Its antioxidant ability is primarily determined by its chemical structure. Specifically, a single hydrogen atom in the OH group of FA can react with free radicals, resulting in the formation of the ferulic acid phenotypic radical (FAPR), which is the end product of the antioxidant ability of FA (Figure 1 [24]). FAPR is highly stable and cannot further initiate or propagate free radical chain reactions [24]. Furthermore, it can only react with another FAPRs to generate dimer curcumin, which contains phenolic hydroxyl groups that also help to eliminate free radicals. This process greatly enhances the free radical scavenging ability of FA [24,25]. Due to its potent antioxidant properties, FA has been extensively researched and used for various applications in different fields. A previous study showed that FA can alleviate oxidative damage caused by H_2_O_2_ to retinal pigment epithelium (RPE) cells [26]. In terms of the germ cells that we are concerned about, FA supplementation can significantly improve human sperm motility, especially in the semen of patients with asthenospermia, which can significantly prolong the duration of sperm motility [27]. Among female germ cells, during the IVM of porcine oocytes, supplementation of 10 μM FA increased the maturation rate of porcine oocytes and improved the cleavage rate and blastocyst formation rate of IVF [28]. However, the effect of FA on bovine oocyte IVM and subsequent embryo development is still unclear. In this experiment, we analyzed the IVM rates of bovine oocytes, the levels of ROS, GSH, and antioxidant enzyme activity in the oocytes, and the development of the consequent early embryos.

In the present study, we hypothesized that supplementing FA during bovine oocyte IVM would enhance the antioxidant capacity of the oocytes, reduce the oxidative stress levels of the oocytes, and improve the oocyte quality as well as subsequent embryo development after in vitro fertilization (IVF) and somatic cell nuclear transfer (SCNT). The results are of great significance for improving bovine embryo production in vitro and for helping to further reveal the antioxidant effects of FA in bovine oocytes.

## 2. Results

### 2.1. Ferulic Acid Promotes the Maturation of the Nuclei and Cytoplasms of Bovine Oocytes In Vitro

The maturation of oocytes involves both nuclear maturation and qualitative maturation, which refers to the ability of the oocytes to undergo meiosis and subsequent development. In our experiments, we assessed the nuclear maturation and qualitative maturation of oocytes by measuring the rates of first polar body extrusion and the potential of subsequent embryo development. Initially, we added varying concentrations of FA (0, 2.5, 5, 10, 20 μM) to the IVM medium to determine the rates of first polar body extrusion in bovine oocytes. The results demonstrated a significant improvement in the rates of first polar body extrusion in bovine oocytes supplemented with 5 μM FA during IVM (Table 1; *p* < 0.05) compared to the control group. Consequently, we selected a concentration of 5 μM as the treatment concentration for subsequent experiments.

**Figure 2:**
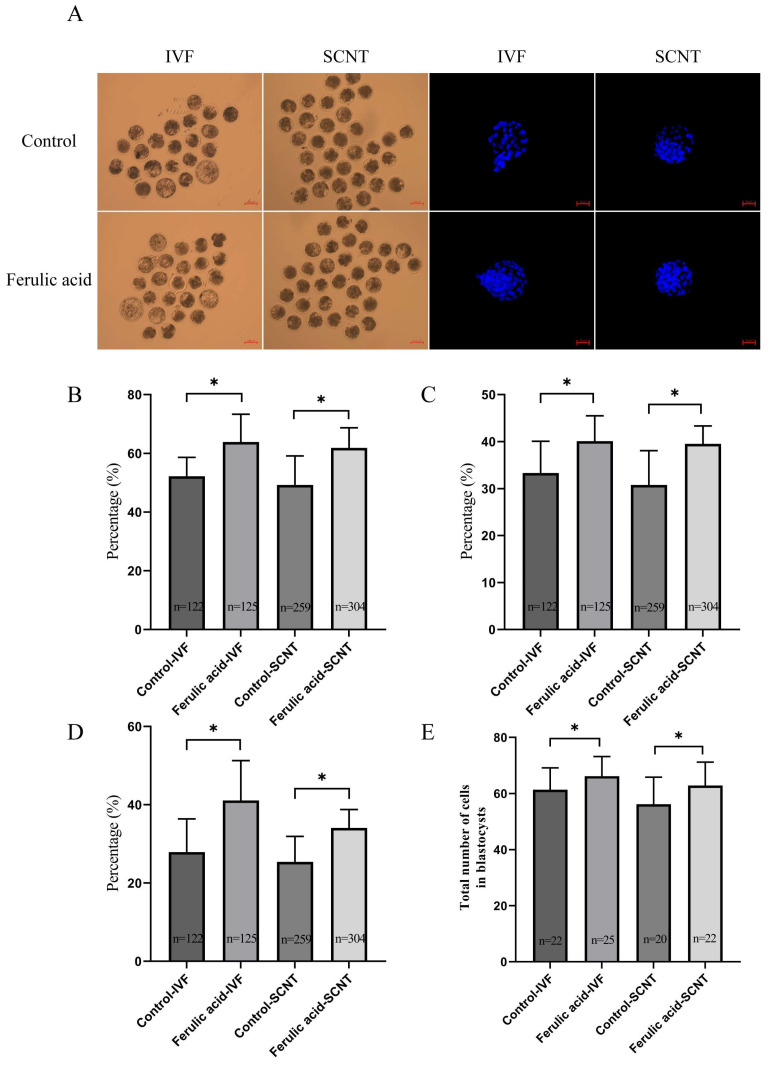
Ferulic acid promotes the maturation of the nucleus and cytoplasm of bovine oocytes in vitro. (**A**) IVF and SCNT embryo development map scale = 100 µm and Hoechst 33342 staining scale = 50 µm for the control group and FA treatment group. (**B**) Cleavage rates of IVF and SCNT embryos in the control group and FA treatment group; * indicates that they significantly differed (*p* < 0.05). (**C**) 8-cell rates of IVF and SCNT embryos in the control group and FA treatment group; * indicates that they significantly differed (*p* < 0.05). (**D**) Blastocyst rates of IVF and SCNT embryos in the control group and FA treatment group; * indicates that they significantly differed (*p* < 0.05). (**E**) The total cells number of IVF and SCNT blastocysts in the control group and FA treatment group; * indicates that they significantly differed (*p* < 0.05).

We next examined whether FA supplementation during the IVM process can improve the developmental competence of bovine oocytes as determined by IVF and SCNT. The results of these experiments indicated a significant enhancement in the cleavage and blastocyst formation rates of embryos that had undergone IVF and SCNT following FA supplementation (Figure 2B–D; *p* < 0.05) compared to those that were not given FA supplementation. Additionally, the total number of cells present in the blastocysts formed by oocytes that had undergone FA supplementation exhibited a significant increase compared to that of oocytes that did not receive FA supplementation (Figure 2E; *p* < 0.05).

### 2.2. Ferulic Acid Supplementation Improves the Quality of Oocytes and the Developmental Potential of Subsequent Embryos

**Figure 3:**
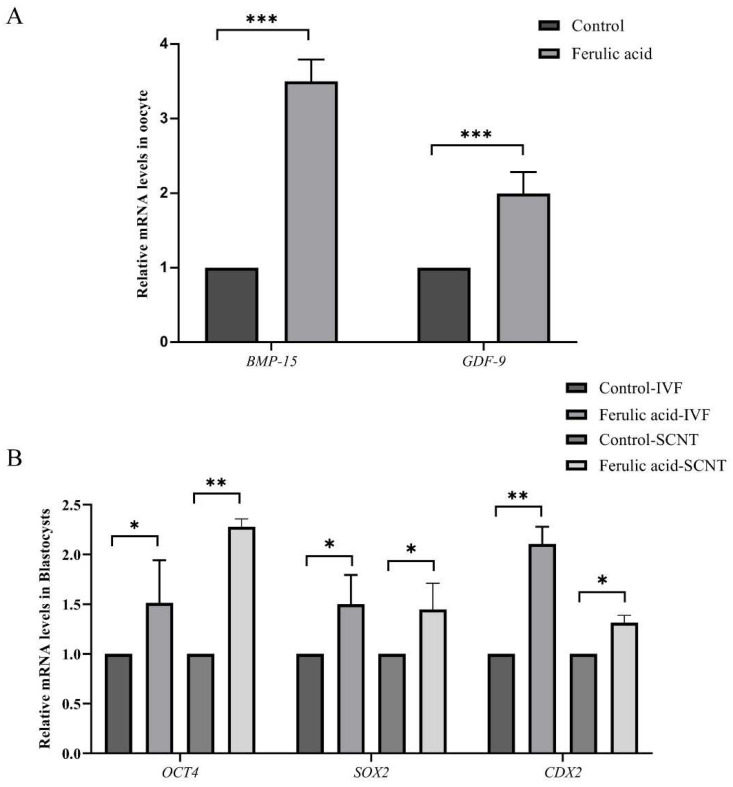
Ferulic acid improves the quality of oocytes and the developmental potential of subsequent embryos. (**A**) The relative mRNA levels of bovine oocyte maturation-related genes (*BMP-15* and *GDF-9*); *** indicates that they significantly differed (*p* < 0.001). (**B**) Relative mRNA expression levels of IVF and SCNT embryo pluripotency-related genes (*OCT4*, *SOX2*, and *CDX2*); ** indicates that they significantly differed (*p* < 0.01), * indicates that they significantly differed (*p* < 0.05).

To elucidate the impact of FA on oocyte quality and the potential for subsequent embryo development, we measured the relative mRNA expression levels of genes associated with the quality of oocytes and the embryonic pluripotency of blastocysts treated with IVF and SCNT. The RT-qPCR results revealed that FA treatment significantly enhanced the relative mRNA expression levels of *GDF-9* and *BMP-15* in oocytes (Figure 3A; *p* < 0.001), as well as the expression of the *SOX2*, *CDX2*, and *OCT4* genes in blastocysts treated with IVF and SCNT (Figure 3B; *p* < 0.05, *p* < 0.01), compared to the untreated control group.

### 2.3. Ferulic Acid Promotes Cumulus Expansion during In Vitro Maturation

**Figure 4:**
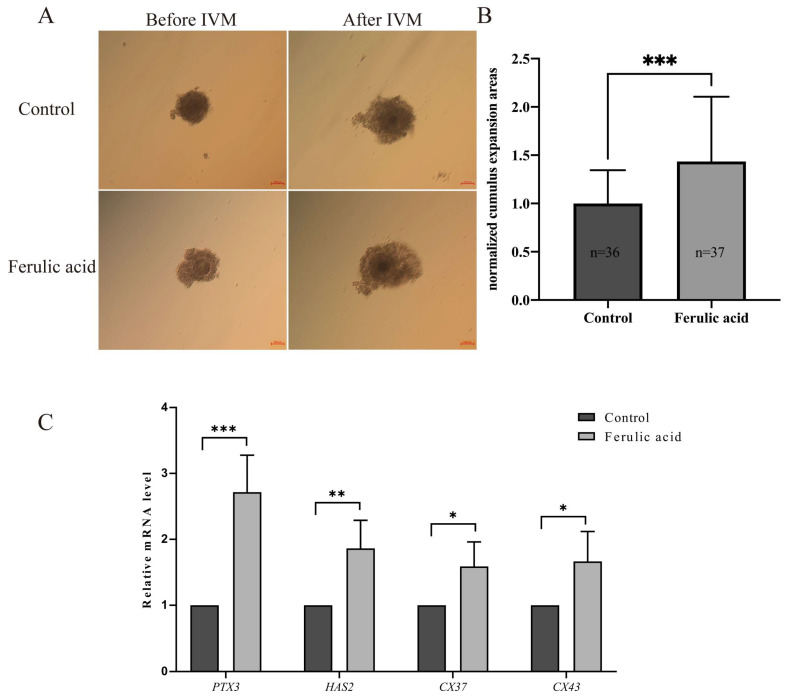
Ferulic acid promotes cumulus expansion during in vitro maturation. (**A**) Representative images of COCs in the control group and ferulic acid group before and after IVM. Scale = 100 μm. (**B**) ImageJ software was used to measure and calculate the statistics of the relative expansion area of cumulus cells; *** indicates that they significantly differed (*p* < 0.001). (**C**) The relative mRNA expression levels of expansion genes (*PTX3*, *HAS2*, *CX37*, and *CX43*) of bovine cumulus cells; * indicates that they significantly differed (*p* < 0.05), ** indicates that they significantly differed (*p* < 0.01), *** indicates that they significantly differed (*p* < 0.001).

The expansion of cumulus cells during IVM is a crucial factor in oocyte maturation and is commonly used as an indicator to evaluate the quality of oocytes [29]. Our results showed a significantly higher expansion area in the treatment group than in the control group (Figure 4B; *p* < 0.001). Moreover, the mRNA expression levels of the cumulus dilatation-related genes *PTX3* and *HAS2*, as well as the cumulus gap junction-related genes *CX37* and *CX43*, were significantly increased in the treatment group compared to the control group (Figure 4C; *p* < 0.05, *p* < 0.01, *p* < 0.001). These results indicate that supplementation with FA during IVM improves the maturation rates and the cumulus expansion areas of bovine oocytes.

### 2.4. Ferulic Acid Improves the Antioxidant Capacity of Bovine Oocytes

**Figure 5:**
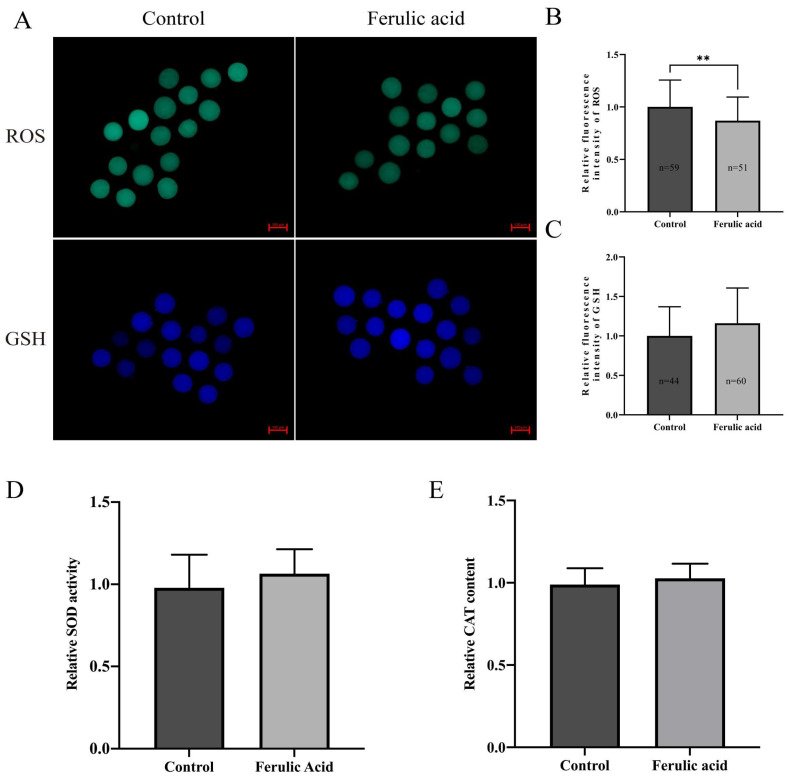
Ferulic acid improves the antioxidant capacity of bovine oocytes. (**A**) The oocytes of the MII stage control group and FA-treated group were exposed to DCHF-DA and CellTracker™ Blue CMF2HC staining, and the intracellular ROS and GSH levels were observed by fluorescence microscopy. Scale = 100 µm. (**B**) Analysis of the relative fluorescence intensity of the ROS signal with ImageJ software; ** indicates that they significantly differed (*p* < 0.01). (**C**) Analysis of the relative fluorescence intensity of the GSH signal with ImageJ software. (**D**) Relative SOD activity in oocytes of the control group and FA treatment group. (**E**) Relative CAT content in oocytes of the control group and FA treatment group.

**Figure 6:**
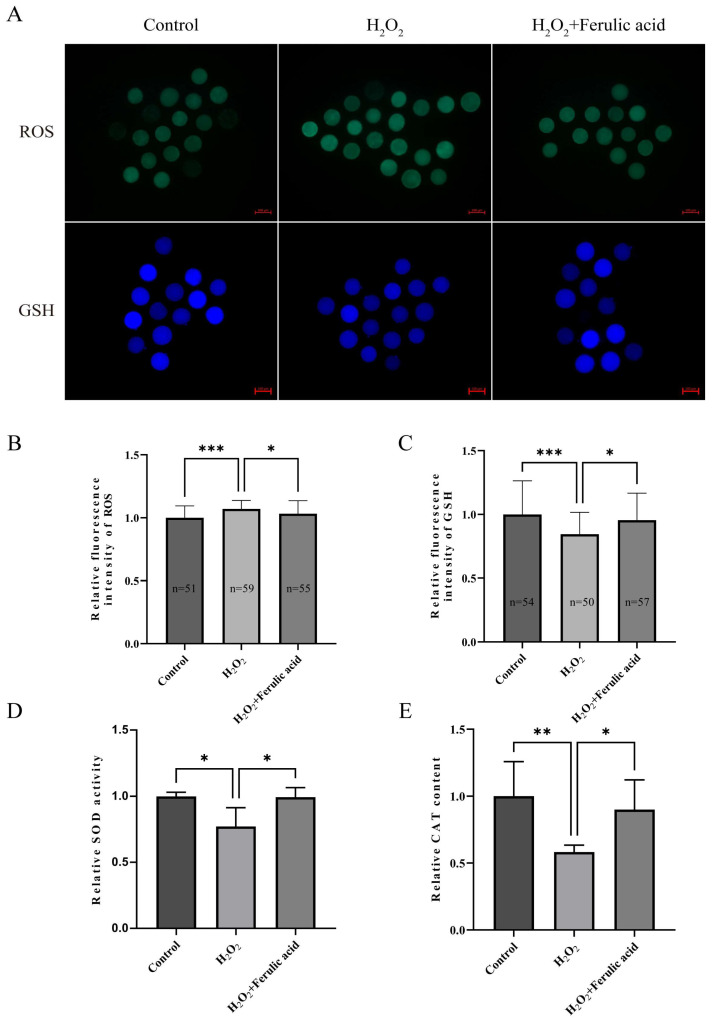
Ferulic acid improves the antioxidant capacity of bovine oocytes treated with H_2_O_2_. (**A**) The oocytes of the MII stage control group, H_2_O_2_ treatment group, and H_2_O_2_-FA co-treatment group were exposed to DCHF-DA and CellTracker™ Blue CMF2HC staining, and the intracellular ROS and GSH levels were observed by fluorescence microscopy. Scale = 100 µm. (**B**) Analysis of the relative fluorescence intensity of the ROS signal with ImageJ software; *** indicates that they significantly differed (*p* < 0.001), * indicates that they significantly differed (*p* < 0.05). (**C**) Analysis of the relative fluorescence intensity of the GSH signal with ImageJ software; *** indicates that they significantly differed (*p* < 0.001), * indicates that they significantly differed (*p* < 0.05). (**D**) Relative SOD activity in oocytes of the control group, H_2_O_2_ treatment group, and H_2_O_2_-FA co-treatment group, * significantly differed (*p* < 0.05). (**E**) Relative CAT content in oocytes of the control group, H_2_O_2_ treatment group and H_2_O_2_-FA co-treatment group; * indicates that they significantly differed (*p* < 0.05), ** indicates that they significantly differed (*p* < 0.01).

Since oxidative stress levels are related to oocyte quality and since FA has a strong antioxidant capacity, we measured the ROS and GSH levels in MII bovine oocytes. A fluorescence intensity analysis showed that the relative ROS levels in bovine oocytes from the group that was treated with FA were significantly lower than those in the control group (Figure 5B; *p* < 0.01). However, there was no significant change in the GSH levels (Figure 5C). To further determine the antioxidant capacity of FA in oocytes, oocytes were cotreated with 200 μM H_2_O_2_ and 5 μM FA during IVM. A fluorescence intensity analysis showed that the ROS levels in the FA treatment group were significantly lower than those in the H_2_O_2_ group (Figure 6B; *p* < 0.05), and the GSH levels were significantly higher in the FA treatment group than those in the H_2_O_2_ group (Figure 6C; *p* < 0.05).

Superoxide dismutase (SOD) and catalase (CAT) are crucial antioxidant enzymes that are present in oocytes. We analyzed the activity of SOD and the content of CAT in MII bovine oocytes and found no significant differences between the control and treatment groups (Figure 5D,E). However, after supplying 200 μM H_2_O_2_ and 5 μM FA to the oocytes during IVM, we observed significantly increased SOD activity and CAT content in the oocytes of the FA treatment group compared to those of the H_2_O_2_ group (Figure 6D,E; *p* < 0.05).

### 2.5. Ferulic Acid Improves the Mitochondrial Function of Bovine Oocytes

**Figure 7:**
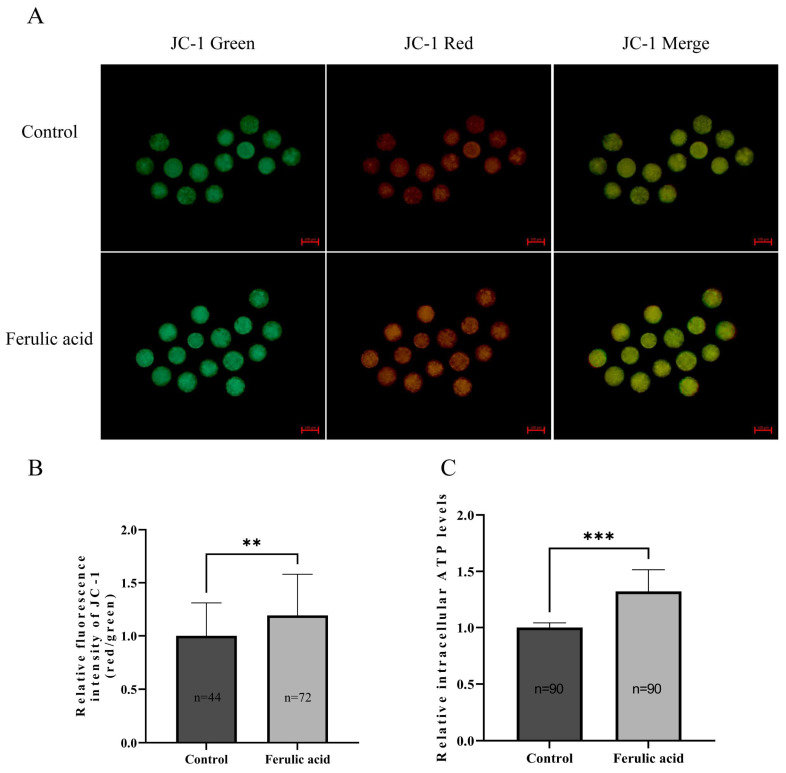
Ferulic acid improves the mitochondrial function of bovine oocytes. (**A**) The oocytes of the FA-treated group in the MII period were exposed to JC-1 staining, and the fluorescence intensity of JC-1 in the cells was observed by fluorescence microscopy. Scale = 100 µm. (**B**) ImageJ analysis of intracellular relative MMP levels; ** indicates that they significantly differed (*p* < 0.01). (**C**) The relative ATP level in oocytes of the control group and FA treatment group; *** indicates that they significantly differed (*p* < 0.001).

Mitochondria are the most important organelles in oocytes and play a decisive role in their maturation. Therefore, the levels of mitochondrial membrane potential (MMP) and adenosine triphosphate (ATP) in bovine oocytes were determined in this study. The results indicated that the levels of MMP and ATP were significantly increased in bovine oocytes that were supplemented with FA compared to those that were not (Figure 7B,C; *p* < 0.01, *p* < 0.001).

## 3. Discussion

FA is a natural antioxidant with a unique chemical structure that enables it to bind to free radicals and form stable phenoxy groups [24]. This study investigated the impact of FA on bovine oocyte maturation. Our study demonstrates that supplementation with FA significantly enhances the maturation rates of oocytes and improves the developmental potential of embryos that are subjected to subsequent IVF and SCNT. Furthermore, FA supplementation effectively mitigates the oxidative stress levels in bovine oocytes and enhances the mRNA expression levels of genes associated with oocyte maturation, cumulus cell expansion, and embryo pluripotency.

The nuclear maturation of oocytes was promoted by FA supplementation of the IVM medium. This finding is consistent with the results of other studies on antioxidants, including a study on bovine oocyte supplementation with nobiletin during IVM [30]. However, we also observed a decrease in the first polar body extrusion rates of oocytes when the FA concentration reached 20 μM. This suggests that, similar to what has been seen with the supplementation of other antioxidants such as melatonin [19], high concentrations of FA in the IVM medium may inhibit oocyte meiosis. In addition, in this study, 5 μM FA increased the first polar body excretion rate of bovine oocytes by about 15%, which is superior to other known antioxidants, such as leonurine [31] or dendrobine [32], which increased the first polar body excretion rate of bovine oocytes by about 9% and 8%, respectively.

The normal development of early embryos serves as the primary indicator in the assessment of the cytoplasmic maturation of oocytes and is an important criterion for evaluating oocyte quality [33,34,35,36]. Our study demonstrated a significant improvement in the cleavage rates and blastocyst formation rates of embryos that were subjected to subsequent IVF and SCNT following FA supplementation. Additionally, we observed a significant increase in the total number of cells present in blastocysts that had undergone IVF and SCNT after FA supplementation. These findings indicate that FA exhibits a similar effect to resveratrol and other antioxidants in facilitating bovine oocyte IVM [19,21]. Besides, during IVM, FA supplementation increased the blastocyst rate of the subsequent IVF and SCNT embryos by about 14% and 11%, respectively, which was higher than that of bovine IVF and SCNT embryos supplemented with other antioxidants. For example, the blastocyst rate of bovine IVF supplemented with Nobiletin increased by about 10% [30], while vitamin C supplementation only increased the blastocyst rate of bovine SCNT by about 2% [37]. The above results show that FA enhances the rates of first polar body extrusion in oocytes and improves the potential for subsequent embryonic development, promotes the maturation of the oocyte nucleus and cytoplasm, and enhances oocyte quality after IVM.

*GDF-9* and *BMP-15*, which are members of the TGF-β family [38,39], are considered to be reliable biomarkers for oocytes maturation [30]. These genes play crucial roles in optimizing the microenvironment during oocyte maturation. *BMP-15* regulates the expression levels of *HAS2*, *PTX3*, and *PTGS2* during oocyte maturation [40,41]. Similarly, *GDF-9* functions by controlling the expression levels of cumulus proliferation-related genes to promote oocyte maturation [41]. Previous studies have demonstrated that the addition of a low concentration of melatonin during IVM not only promotes oocyte maturation but also significantly increases the mRNA expression levels of *GDF-9* and *BMP-15* in oocytes [42,43]. The experiments in this study revealed a significant increase in the relative mRNA expression levels of *GDF-9* and *BMP-15* in bovine oocytes after FA supplementation compared to those that did not receive FA supplementation. This finding suggests that the addition of FA during IVM can effectively enhance the maturation rates and quality of bovine oocytes.

The expression levels of *OCT4*, *SOX2*, and *CDX2* within blastocyst cells determine the developmental capacity of early-stage embryos. *OCT4* and *SOX2* are typically expressed during the activation phase of the zygotic genome and work closely to maintain pluripotency in embryonic stem cells [44,45,46]. Studies have shown that knocking out the *OCT4*, *SOX2*, or *CDX2* genes can lead to abnormal embryo development [47,48,49]. In these experiments, the mRNA expression levels of *OCT4*, *CDX2*, and *SOX2* in the blastocysts that had undergone IVF and SCNT were significantly upregulated after supplementation with FA compared to the expression levels of those that did not receive FA supplementation. This indicates that FA can improve the IVM quality of bovine oocytes and ultimately enhance the developmental capacity of early-stage embryos. A similar result was obtained in a previous study. Rivolta et al. found that supplementation with trace amounts of DMSO during bovine oocyte IVM significantly increased the relative mRNA expression levels of *OCT4*, *SOX2*, *CDX2*, and *SOD1* in blastocysts compared to those that did not receiver DMSO supplementation [50].

The dilation of cumulus cells plays an important role in oocyte meiosis [51,52]. Our results show that supplementation with 5 μM FA significantly increases the expansion area of cumulus cells during IVM. Previous studies have shown that high levels of *HAS2* and *PTX3* in cumulus cells can improve the maturation rate of oocytes and the subsequent embryo development ability. [53,54]. Additionally, another study showed that cumulus cells transfer molecules to oocytes through gap junctions to aid in oocyte maturation. *CX43* and *CX37* are two genes that encode isoforms of gap junction proteins that have the most significant roles in regulating oocyte development and maturation [55]. Therefore, we investigated the mRNA expression levels of these four genes (*PTX3*, *HAS2*, *CX43*, and *CX37*) in cumulus cells and observed that FA supplementation significantly increased their expression levels. Similar changes in mRNA expression levels were observed with the supplementation of epigallocatechin-3-gallate, an unsaturated acid that is structurally related to FA [56]. In addition, in the process of oocyte development, it is necessary to rely on cumulus cells to provide small molecular nutrients such as glucose metabolites and amino acids to meet the needs of oocytes for maturation [52]. In the process of the IVM of oocytes, cumulus cells will also undergo oxidative stress, which will lead to apoptosis. Supplementing antioxidants such as melatonin can eliminate excess ROS in cumulus cells, thus promoting cumulus cell expansion and improving the quality of the IVM of oocytes [57]. This may also be the reason why FA supplementation has a positive effect on the quality of the IVM of oocytes.

During IVM, oocytes are exposed to a relatively hyperoxic environment [58]. The excessive accumulation of ROS is the main cause of oxidative stress in oocytes. Therefore, supplying antioxidants to eliminate excess ROS in oocytes is an effective way to alleviate oxidative stress. A previous study found that supplementing the diets of diabetic rats with FA significantly reduces the ROS levels in their renal cells [59]. Additionally, FA has been shown to alleviate myocardial cell injury caused by H_2_O_2_-induced oxidative stress [60] by scavenging ROS. In our experiments, FA played the same role. Supplementation with FA during IVM effectively reduced the intracellular ROS accumulation in bovine oocytes regardless of the H_2_O_2_ exposure. Furthermore, oocytes maintain a dynamic balance of ROS production and clearance through the activities of antioxidant molecules such as GSH, CAT, and SOD [61,62,63]. Therefore, the contents of these antioxidant molecules in oocytes are an essential indicator for assessing the antioxidant capacity of oocytes [20,64]. Glutathione (GSH) is the most abundant antioxidant in cells, and it can eliminate intracellular active oxygen through the mutual conversion of reduced GSH and oxidized glutathione disulfide (GSSG) [65]. Studies have shown that GSH plays a critical role in oocyte maturation and embryo development, and high levels of GSH result in the production of higher-quality embryos [65,66]. Similarly, CAT and SOD are important antioxidant molecules in oocytes that can remove both superoxide anions and H_2_O_2_ [67,68]. Studies have shown that high-quality oocytes have higher levels of CAT and SOD than low-quality oocytes [69,70,71,72]. In this study, we found that FA supplementation significantly augmented the increases in GSH, CAT, and SOD levels that are initially caused by H_2_O_2_ exposure in bovine oocytes. Notably, in our study, it was found that FA supplementation alone could not improve the activity and content of antioxidant molecules (GSH, SOD, CAT) in bovine oocytes. We believe that this is because FA itself has phenolic hydroxyl groups which can directly capture free radicals to achieve the purpose of scavenging ROS [24]. When oocytes are stimulated by H_2_O_2_, FA can maintain the level of antioxidant molecules and protect the quality of oocytes from damage. These findings are consistent with previous studies. FA supplementation alone cannot improve the levels of GSH, SOD, and CAT in human retinal cells and HEK293 cells, but after co-treatment with H_2_O_2_, the levels of antioxidant molecules in cells are significantly improved [26,73,74,75]. These results show that FA can eliminate ROS in oocytes and reduce the consumption of antioxidant molecules in oocytes when exposed to H_2_O_2_, which can alleviate the oxidative stress in oocytes and improve the quality of oocytes.

Mitochondria are a primary source of intracellular ROS [76]. According to previous studies, ROS can cause mitochondrial dysfunction, resulting in oxidative stress in oocytes, which leads to a decrease in the quality of oocytes and the subsequent embryos [77,78]. Multiple studies have demonstrated that mitochondrial dysfunction can result in reduced oocyte quality and impaired embryonic development [64,78,79]. To assess the mitochondrial function in oocytes, researchers often measure MMP and ATP levels [80,81]. In this study, we analyzed these two indicators and found that FA supplementation led to significant increases in both MMP and ATP levels, which is consistent with prior research [82,83]. These results suggest that FA supplementation can improve the mitochondrial function in oocytes and ultimately enhance oocyte quality.

These results suggest that supplementation with FA during the IVM of bovine oocytes can enhance oocyte quality and improve the developmental potential of subsequent embryos. However, the molecular mechanism underlying this outcome still needs be further explored.

## 4. Materials and Methods

### 4.1. Ethics Statement

All animal studies were reviewed and approved by the Animal Care and Use Committee of Jilin University.

### 4.2. Chemicals

Unless otherwise stated, all chemicals were purchased from Sigma (St. Louis, MO, USA).

### 4.3. Collection and In Vitro Maturation of Oocytes

Bovine ovaries were obtained from a slaughterhouse, stored in normal saline solution containing 1% penicillin–streptomycin solution (P/S) at 37 °C, and shipped to the laboratory within 4 h. Follicle fluid was aspirated from the 2–8 mm antral follicles on the ovarian surface using a 10 mL syringe with a 12-gauge needle. After precipitation, the supernatant was discarded, and the precipitate was mixed with HEPES. The cumulus–oocyte complexes (COCs) surrounded by more than three layers of cumulus cells were collected under a microscope. The 40 COCs were washed three times and transferred to 500 μL droplets of IVM medium covered with mineral oil. They were then placed at 38.5 °C with 5% CO_2_ under humidified air and cultured for 22–24 h to achieve maturity. COCs in each group were repeatedly cultured three times, and the first polar body excretion rate was counted, respectively. The IVM media contained TCM199, FBS (10% *v*/*v*), P/S (1% *v*/*v*), estradiol (2 μg/mL), epidermal growth factor (EGF, 10 ng/mL), follicle-stimulating hormone (FSH, 10 IU/mL), luteinizing hormone (LH, 10 IU/mL), and sodium pyruvate (100 mM). The FA was diluted with DMSO to create solutions with concentrations of 2.5, 5, 10, and 20 M. Before IVM, we diluted the concentrated solutions and IVM medium at a ratio of 1:1000. FA at a final concentration of 2.5, 5, 10, or 20 μM was administered during IVM. In the H_2_O_2_ treatment experiment, we selected H_2_O_2_ with a concentration of 200 μM to induce oxidative stress in oocytes according to the previous literature [84], and treated them with FA with a concentration of 5 μM.

### 4.4. Statistics of Cumulus Expansion Area

A single COC was placed in 10 μL IVM medium covered with mineral oil or the FA treatment group, and then transferred to a constant temperature carbon dioxide incubator with 39 °C, 5% CO_2_, and saturated humidity for 20 h, and then the COCs was photographed again. The area of the two pictures was measured by Image J software, and the COC’s area difference ratio before and after IVM was calculated.

### 4.5. In Vitro Fertilization and In Vitro Culture of Embryos

We supplemented 5 μΜ FA during IVM. After IVM, the oocytes were washed three times with BO-IVF medium (IVF Bioscience, Falmouth, UK). Then, 5–8 oocytes per group were transferred to 40 μL of IVF medium droplets and incubated under mineral oil in a humidified environment containing 5% CO_2_ at 39 °C. The frozen bovine sperm was thawed for 15 s in a water bath at 37 °C and purified using Percoll gradient centrifugation. Following purification, the sperm density was determined using a hemocytometer, and the sperm density was adjusted to 1 × 10^6^ spermatozoa/mL. The spermatozoa and oocytes were cultured in BO-IVF medium for 22 h at 39 °C in 5% CO_2_ humidified air. After IVF, the zygotes were cultured in BO-IVC medium (IVF Bioscience, Falmouth, UK) for 8 days at 39 °C in 5% CO_2_ humidified air. The culture medium was changed every day throughout the culture period. The blastocyst formation rate was counted on day 7.

### 4.6. Somatic Cell Nuclear Transfer and In Vitro Culture of Embryos

We supplemented 5 μΜ FA during IVM. After IVM, the cumulus cells were removed, and the oocytes were subjected to SCNT procedures, following the protocol used in our previous studies [85]. After 22 h of IVM, cumulus cells were removed from the oocyte by gentle pipetting in TL-HEPES supplemented with 1 mg/mL hyaluronidase. The oocyte nucleus was removed along with the first polar body by blind aspiration, and somatic cells were injected into the perivitelline space of the oocyte. Membrane fusion was induced by applying an alternating current field with 2 V cycling at 1 MHz for 2 s, followed by a DC pulse of 200 V/mm for 20 μs, using a cell fusion generator (NEPA GENE ECFG21, Chiba, Japan). Following fusion, the reconstructed embryos were placed in IVC medium for 30 min before activation. All the reconstructed embryos were activated in 5 μM ionomycin for 5 min, followed by 4 h of exposure to 1.9 mM 6-dimethylpyridine in IVC medium. After activation, the reconstructed embryos were cultured in IVC medium for 8 days at 39 °C in 5% CO_2_ humidified air. The culture medium was changed every day throughout the culture period. The blastocyst formation rate was counted on day 7.

### 4.7. Determination of ROS, GSH and MMP Levels in Bovine Oocytes

The ROS levels, GSH levels, and MMP levels in the oocytes were measured using a ROS detection kit (Beyotime, Shanghai, China), GSH level detection kit (Thermo Fisher Scientific, Waltham, MA, USA), and JC-1 assay kit (Thermo Fisher Scientific, Waltham, MA, USA), respectively. To determine the ROS levels in the oocytes, cumulus cell-free oocytes were cultured in PBS-PVA medium containing DCFH-DA (10 μM) for 30 min. To determine the GSH levels in the oocytes, cumulus cell-free oocytes were cultured in PBS-PVA medium containing CMF2HC (10 μM) for 30 min. To determine the MMP levels in the oocytes, cumulus cell-free oocytes were cultured in PBS-PVA medium containing JC-1 (2 μM) for 30 min. After incubation, the dye was washed off with PBS-PVA medium, and the fluorescence intensity of the oocytes was detected and captured using a fluorescence microscope and then saved as a Tiff file. The fluorescence signal intensity of each group of oocytes was analyzed using ImageJ software.

### 4.8. Determination of ATP Levels in Bovine Oocytes

An ATP assay kit (Beyotime, Shanghai, China) was used to detect the ATP level in oocytes. MII-stage oocytes from each group were collected, lysed with 200 μL of lysis buffer, and then centrifuged at 12,000 rpm for 5 min at 4 °C. The supernatant was collected and placed on ice for measurement. Next, 20 μL of the supernatant and 100 μL of ATP working solution were added to a 96-well opaque plate and analyzed using a microplate reader.

### 4.9. RNA Extraction, Reverse Transcription and RT–qPCR

Bovine embryos’ and cumulus cells’ RNA was extracted using the Micro Scale RNA Isolation Kit (Thermo Fisher Scientific, Waltham, MA, USA) extraction method. First-strand cDNA was synthesized using MonScript™ RTIII ALL-in-One Mix with a dsDNase kit (Monad, Wuhan, China). RT–qPCR was performed using the MonAmp™ ChemoHS qPCR Mix kit (Monad, Wuhan, China). The primers used for RT–qPCR were designed using Primer Premier 5.0 software. The RT–qPCR primers for each gene are shown in Table 2. The relative mRNA expression levels were determined using the 2^−△△Ct^ method and normalized to *GAPDH*.

### 4.10. Determination of SOD Activity and CAT Content in Bovine Oocytes

The Catalase Activity Assay Kit (Solarbio, Beijing, China) and Total Superoxide Dismutase Assay Kit with WST-8 (Beyotime, Shanghai, China) were used to determine the activity of SOD and the content of CAT in oocytes. To determine the CAT content, MII-stage oocytes from each group were collected, and 15 μL of extraction solution was added. The cells were lysed using ultrasound (power 200 W, ultrasound 3 s, interval 10 s, repeated 30 times). The supernatant was collected after centrifugation at 8000× *g* for 10 min at 4 °C and placed on ice for measurement. 10 μL of supernatant and 190 μL of CAT working solution were added to a 96-well opaque plate, and the sample was analyzed using a microplate reader. To determine the SOD activity, MII-stage oocytes from each group were collected, and 25 μL of SOD sample preparation solution was added and mixed well by pipetting. The cells were then centrifuged at 12,000× *g* for 5 min at 4 °C. The supernatant was collected and placed on ice for measurement. Then, 20 μL of supernatant, 160 μL of WST-8/enzyme working solution, and 20 μL of reaction starter working solution were added to a 96-well opaque plate. A blank control was set according to the instructions, and the sample was analyzed using a microplate reader.

### 4.11. Statistical Analysis

SPSS was utilized to analyze all of the data. We employed independent t tests or one-way analysis of variance (ANOVA) for intergroup comparisons. Values are expressed as the mean ± standard deviation (SD). A *p* value of less than 0.05 was considered statistically significant. Each experiment was conducted with three or more replicates.

## 5. Conclusions

**Figure 8:**
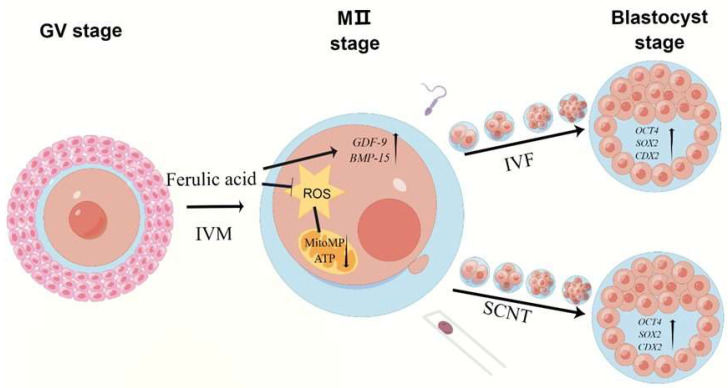
Diagram of ferulic acid enhancing bovine oocyte maturation and subsequent early embryo potential. During the IVM of bovine oocytes, supplementation with FA effectively reduced the level of intracellular ROS, resulting in improved mitochondrial function. Meanwhile, FA supplementation positively influenced the mRNA levels of *GDF-9* and *BMP-15* in oocytes, leading to enhanced oocyte quality. Moreover, it increased the mRNA levels of *OCT4*, *SOX2*, and *CDX2* in IVF and SCNT blastocysts, thereby augmenting the developmental capacity of early embryos. Figdraw was used to make the diagram.

In summary, our results demonstrated that supplementation with FA improved the IVM rates of bovine oocytes and the blastocyst formation rates of embryos that underwent IVF and SCNT. Moreover, it alleviated the cellular oxidative stress levels in bovines during IVM, leading to an improved quality of bovine oocytes and early embryos (Figure 8). Further studies are needed to elucidate the underlying mechanisms and potential application values of FA in promoting oocyte maturation in assisted reproductive technology.

## Figures and Tables

**Table 1:** In vitro maturation parameters of bovine oocytes in the presence of ferulic acid.

Parameters Evaluated	Total Number of Oocytes *n*	First Polar Body Extrusion *n* (%)
Control	122	69 (56.55 ± 4.116)
2.5 μM Ferulic acid	102	58 (56.86 ± 2.318)
5 μM Ferulic acid	125	89 (71.2 ± 3.838) *
10 μM Ferulic acid	97	57 (58.76 ± 6.052)
20 μM Ferulic acid	145	78 (53.79 ± 2.775)

*n*: number of oocytes assigned per group. Control: oocytes cultured in the presence of IVM medium supplemented with 0.01% DMSO; 2.5 μM Ferulic acid, 5 μM Ferulic acid, 10 μM Ferulic acid, 20 μM Ferulic acid: oocytes cultured in presence of IVM medium supplemented with 2.5, 5, 10, and 20 μM ferulic acid, respectively. Data are the mean ± SD. * represents a significant difference from the control group.

**Table 2:** PCR primer sequences.

Gene Symbol	Primer	Primer Sequences (5′–3′)	Accession Number (NCBI)
*GAPDH*	F-primer	TTCAACGGCACAGTCAAGG	NM_001034034
	R-primer	ACATACTCAGCACCAGCATCAC	
*PTX3*	F-primer	CACAGGTCATGTTGTTCCTGAG	NM_001076259.2
	R-primer	CAGATATTGAAGCCTGTGAGTCTG	
*HAS2*	F-primer	TCTCTAGAAACCCCCATTAAGTTG	NM_174079.3
	R-primer	ATCTTCCGAGTTTCCATCTATGAC	
*CX43*	F-primer	CTTTCGTTGTAACACTCAACAACC	NM_174068.2
	R-primer	GTAGAACACATGAGCCAGGTACAG	
*CX37*	F-primer	AGCCCGTGTTTGTGTGCCAG	NM_001083738.1
	R-primer	ACCAGGGAGATGAGTCCGACCA	
*OCT4*	F-primer	GGAGAGCATGTTCCTGCAGTGC	NM_174580
	R-primer	ACACTCGGACCACGTCCTTCTC	
*CDX2*	F-primer	CCTGTGCGAGTGGATGCGGAAG	XM_871005
	R-primer	CCTTTGCTCTGCGGTTCT	
*SOX2*	F-primer	CGAGTGGAAACTTTTGTCCG	NM_001105463
	R-primer	GGTATTTATAATCCGGGTGTT	
*GDF-9*	F-primer	TCCAGAACCTTGTCAATGAG	NM_001031752.1
	R-primer	GGGCAATCATACCCTCATAC	
*BMP-15*	F-primer	GGACCCCTAAATCCAACAGA	NM_174681.2
	R-primer	ACAGTAACACGATCCAGGTT	

## Data Availability

The dataset generated and/or analysed during the current study is available from the corresponding author on reasonable request.
